# Retraction: Long non-coding RNA AK096174 promotes cell proliferation and invasion in gastric cancer by regulating WDR66 expression

**DOI:** 10.1042/BSR-2018-0277_RET

**Published:** 2023-03-02

**Keywords:** AK096174, gastric cancer, invasion, long noncoding RNA, WD repeat-containing protein

This Retraction follows an Expression of Concern relating to this article previously published by Portland Press.

This article is being retracted from *Bioscience Reports* at the request of the Editor-in-Chief and the Editorial Board following receipt of a notification from a reader, alerting the Editorial Board to incorrect nucleotide sequence reagents listed. The errors in this article were identified using The Seek & Blastn tool by Labbé et al. 2019 (https://doi.org/10.1371/journal.pone.0213266).

The authors have been contacted with regards to the retraction, and have not responded to the Journal's queries and the concerns raised. Given the extent of the issues raised, the Editorial Board stand by the decision to retract the article.

